# Stress Leads to Contrasting Effects on the Levels of Brain Derived Neurotrophic Factor in the Hippocampus and Amygdala

**DOI:** 10.1371/journal.pone.0030481

**Published:** 2012-01-17

**Authors:** Harini Lakshminarasimhan, Sumantra Chattarji

**Affiliations:** National Centre for Biological Sciences, Tata Institute of Fundamental Research, Bangalore, India; Chiba University Center for Forensic Mental Health, Japan

## Abstract

Recent findings on stress induced structural plasticity in rodents have identified important differences between the hippocampus and amygdala. The same chronic immobilization stress (CIS, 2h/day) causes growth of dendrites and spines in the basolateral amygdala (BLA), but dendritic atrophy in hippocampal area CA3. CIS induced morphological changes also differ in their temporal longevity- BLA hypertrophy, unlike CA3 atrophy, persists even after 21 days of stress-free recovery. Furthermore, a single session of acute immobilization stress (AIS, 2h) leads to a significant increase in spine density 10 days, but not 1 day, later in the BLA. However, little is known about the molecular correlates of the differential effects of chronic and acute stress. Because BDNF is known to be a key regulator of dendritic architecture and spines, we investigated if the levels of BDNF expression reflect the divergent effects of stress on the hippocampus and amygdala. CIS reduces BDNF in area CA3, while it increases it in the BLA of male Wistar rats. CIS-induced increase in BDNF expression lasts for at least 21 days after the end of CIS in the BLA. But CIS-induced decrease in area CA3 BDNF levels, reverses to normal levels within the same period. Finally, BDNF is up regulated in the BLA 1 day after AIS and this increase persists even 10 days later. In contrast, AIS fails to elicit any significant change in area CA3 at either time points. Together, these findings demonstrate that both acute and chronic stress trigger opposite effects on BDNF levels in the BLA versus area CA3, and these divergent changes also follow distinct temporal profiles. These results point to a role for BDNF in stress-induced structural plasticity across both hippocampus and amygdala, two brain areas that have also been implicated in the cognitive and affective symptoms of stress-related psychiatric disorders.

## Introduction

Accumulating evidence indicates that the same behavioral stress, such as 10 days of chronic immobilization stress (2 hours/day), can elicit contrasting patterns of structural plasticity in the rat hippocampus and amygdala simultaneously [1]–[3]. In addition to being essential components of the neural circuitry mediating stress responses, these two brain areas have also been implicated in the cognitive and affective symptoms of stress-related psychiatric disorders [4], [5]. Earlier studies revealed that repeated or chronic stress causes dendritic atrophy in CA3 pyramidal neurons of the rodent hippocampus [6]. In the basolateral amygdala (BLA), by contrast, chronic stress triggers the opposite effect by strengthening the structural basis of synaptic connectivity through dendritic growth and spinogenesis [3]. Stress-induced dendritic remodeling in these two brain areas differ not only in polarity, but also in terms of their temporal persistence. For instance, exposure to 10 days of chronic immobilization stress elicits dendritic hypertrophy in BLA principal neurons that lasts till at least 21 days after the termination of stress [7]. Hippocampal CA3 atrophy, on the other hand, is reversible within the same period of post-stress recovery [1], [7]. Interestingly, the unique temporal features of stress-induced changes in the BLA are not limited to chronic stress alone. The temporal pattern of structural plasticity in the BLA can also be modulated by the duration of the stressor. A much shorter duration of the same stress, such as a single 2 h episode of immobilization, that fails to affect spine density or dendritic arborization one day later, leads to a significant increase in spine density ten days later [8]. Together, these studies have helped identify novel features of stress-induced plasticity in the amygdala that are quite distinct from those observed in the hippocampus.

Although little is known about molecular mechanisms underlying these contrasting effects of stress, previous studies in the hippocampus provide valuable leads. For example, the same chronic stress that elicits hippocampal dendritic atrophy also reduces levels of the neurotrophin brain-derived neurotrophic factor (BDNF) in the rodent hippocampus [9], [10]. Conversely, chronic administration of antidepressants prevents stress-induced decrease in BDNF levels and dendritic atrophy in the hippocampus [9], [11]. Together these and other findings have contributed to the “neurotrophic hypothesis”, which states that symptoms associated with stress-related disorders such as depression are a result of decreased neurotrophic support, and conversely, that increasing neurotrophic support would lead to the correction of these symptoms [12], [13]. This hypothesis has received support from several studies including a report that direct BDNF infusion into the rodent hippocampus produces antidepressant effects [14], [15]. Also, transgenic overexpression of the neurotrophin BDNF has antidepressant effects and prevents chronic stress-induced hippocampal atrophy in mice [16]. Interestingly, in the same transgenic mice, overexpression of BDNF also causes spinogenesis in the BLA. Moreover, BLA spinogenesis is also triggered by chronic stress in control mice but is occluded by BDNF overexpression, thereby suggesting a role for BDNF signaling in stress-induced plasticity in the amygdala. These findings, in turn, are consistent with the significant body of evidence establishing a role for BDNF as a potent regulator of morphological plasticity of dendrites in various brain regions [17]. Therefore, in the present study we test the prediction that if BDNF plays a key role in stress-induced structural plasticity across both hippocampus and amygdala, then the divergent effects of stress should also be manifested as differential patterns of BDNF expression in these two brain areas.

## Materials and Methods

### Animals

Eight week-old (adult) male Wistar rats (National Centre for Biological Sciences, Bangalore, India) were housed in groups of 2 or 3 in a standard 14 h light and 10 h dark schedule (lights on at 7:00 A.M) with *ad libitum* access to food and water. All animal care and experimentation procedures were approved by the Institutional Animal Ethics Committee, National Centre for Biological Sciences (Approval No: SC-5/2009) and Committee for the Purpose of Control and Supervision of Experiments on Animals, Government of India (Registration No: 109/CPCSEA).

### Stress protocol and experimental design

**Figure 1:**
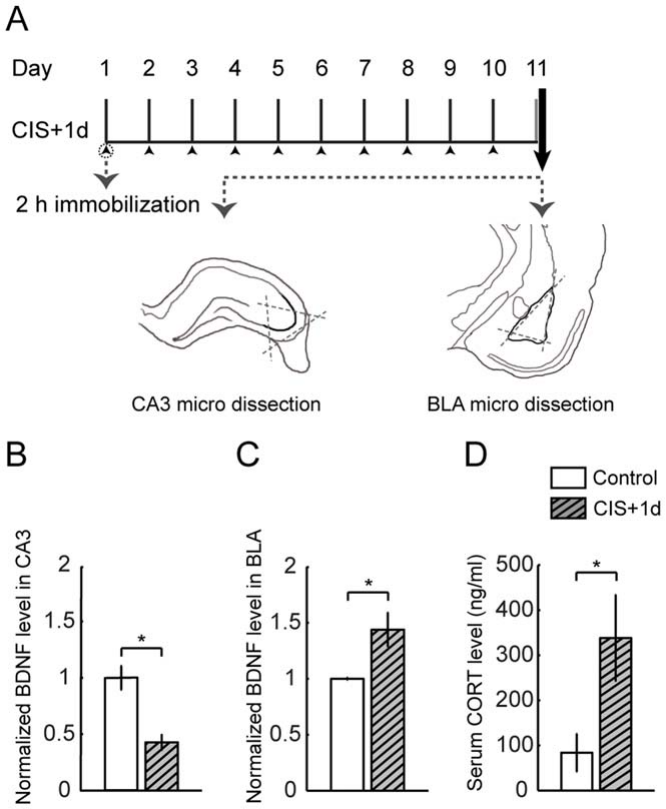
Chronic stress regulates BDNF expression in CA3 and BLA in contrasting manner. Data are presented as mean ± SEM. **A** Experimental design of chronic stress (CIS+1d) followed by micro dissection of area CA3 from transverse hippocampal sections or BLA from coronal sections. **B** BDNF expression in area CA3 is decreased after chronic stress (Control, n = 5; CIS+1d, n = 5), **C** Chronic stress up regulates BDNF expression in BLA (Control, n = 6; CIS+1d, n = 8). Chronic stress induced changes in BDNF expression profile is normalized to respective control level. **D** Serum corticosterone levels are up regulated after chronic stress in comparison to controls (Control, n = 5; CIS+1d, n = 6). Significant differences *, p<0.05; Unpaired, two-tailed, Student's t-test was used to compare stress and control groups.

**Figure 2:**
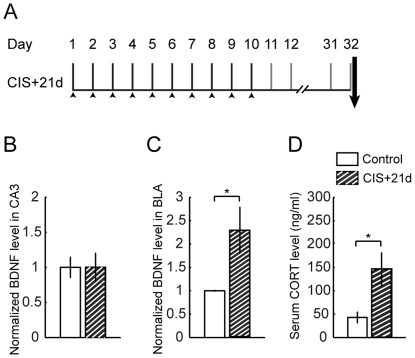
Contrasting profiles of BDNF in CA3 and BLA in response to recovery from chronic stress. Data are presented as mean ± SEM **A** Experimental design of 21 days of recovery from chronic stress paradigm (CIS+21d). **B** BDNF expression in area CA3 comes back to control levels post recovery from chronic stress (Control, n = 6; CIS+21d, n = 6), **C** Chronic stress induced up regulation in BDNF expression in BLA persists even after twenty one days of recovery (Control, n = 4; CIS+21d, n = 6). BDNF expression profile is normalized to respective control level. **D** The up regulation of serum corticosterone levels persists even after recovery from chronic stress (Control, n = 7; CIS+21d, n = 6). Significant differences *, p<0.05; Unpaired, two-tailed, Student's t-test was used to compare stress and control groups.

**Figure 3:**
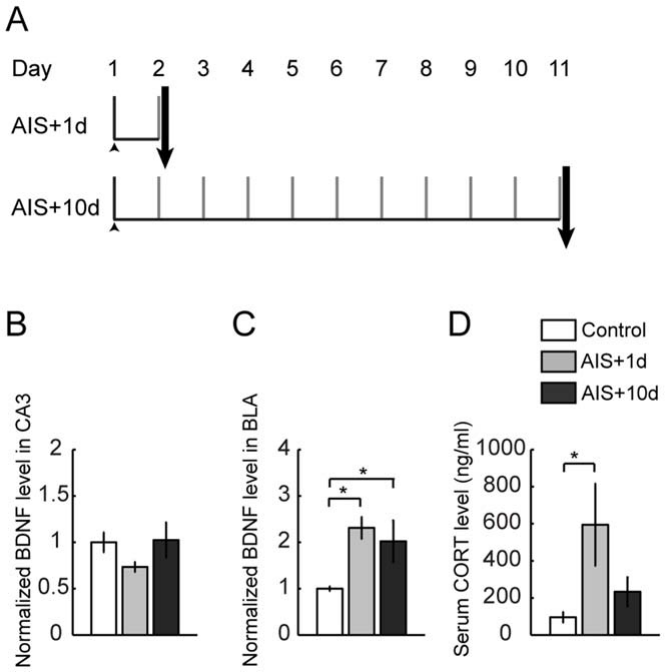
Acute stress modulates BDNF profile differentially in CA3 and BLA. Data are presented as mean ± SEM. **A** Experimental design of acute stress (AIS+1d) and recovery for 10 days from acute stress (AIS+10d). **B** BDNF expression in area CA3 in response to acute stress. Acute stress does not change the BDNF levels in CA3 (Control, n = 9; AIS+1d, n = 9; AIS+10d, n = 6, F_(2,21)_ = 2.11, p>0.05). **C** Acute stress up regulates BDNF expression in BLA and this up regulation persists even after recovery (Control, n = 6; AIS+1d, n = 5; AIS+10d, n = 6, F_(2,14)_ = 4.82, p<0.05). BDNF expression profile was normalized to respective control levels and evaluated by Fisher's *post-hoc*. **D** Effect of acute stress and recovery from acute stress on serum corticosterone levels (Control, n = 11; AIS+1d, n = 5; AIS+10d, n = 6, Kruskal-Wallis ANOVA, χ^2^_(2)_ = 10.01, p<0.05, Mann-Whitney *post-hoc,* p<0.05). Significant differences *, p<0.05.

The behavioral stress protocol has been described elsewhere [3], [7], [8]. Briefly, rats were randomly assigned to experimental groups – controls or different intensities of stress. Rats were subjected to chronic immobilization stress (CIS) or acute immobilization stress (AIS). Subsequently random subsets of the stressed rats were allowed to recover for 21 days from CIS and 10 days from AIS. Stress consisted of complete immobilization for 2 h per day (between 10 AM and noon) in rodent immobilization bags without access to either food or water, for 10 consecutive days in case of CIS (CIS+1d) and a subset of these rats were allowed to recover for 21 days (CIS+21d) (Figure 1A and 2A). AIS consisted of a single immobilization session of 2 h, after which either 1 day (AIS+1d) or 10 days (AIS+10) of recovery later animals were sacrificed (Figure 3A). Day 1 marks the beginning of the experiment while arrow indicates the end point.

### Body weights

The net change in weights of rats between the beginning and end points of the experiments was divided by the starting weight and multiplied by 100 to calculate percentage gain in body weights.

### Corticosterone measurements

Rats were weighed and sacrificed by rapid decapitation under deep anesthesia (halothane) and trunk blood was collected within 3 minutes for each animal; all samples were collected between 10 AM and 1 PM. Samples were centrifuged at 16000 rcf for 5 min at 4°C, and serum was stored at −80°C. Serum corticosterone level was determined by using Correlate - EIA kit according to the manufacturer's instructions (Assay designs). Absorbance at 405 nm was determined by microplate reader (Biorad). Corticosterone concentration (ng/ml) was determined using the standard curve. Anesthesia, decapitation and blood collection was conducted rapidly (within 3′) in an adjacent room in order to minimize stress effects and keep the time to blood collection standardized.

### Tissue collection and BDNF measurements

Basolateral amygdala (BLA) was micro dissected from 400 µm thick coronal sections obtained from McIlwain Tissue Chopper. Area CA3 was dissected from 400 µm thick transverse hippocampal sections obtained using the chopper (Figure 1A). Tissue was lysed in the presence of protease inhibitor cocktail (Roche). BDNF was estimated using BDNF Emax immunoassay kit (Promega) according to manufacturer's protocol. Protein levels were estimated using BCA kit (Pierce). Levels of BDNF were normalized to protein levels.

### Data analysis

Percentage changes in body weight gain were compared between control and experimental groups using unpaired Student's t test assuming unequal variances. Corticosterone measurements and BDNF data were analyzed using unpaired Student's t test assuming unequal variances in the case of CIS and CIS+21d. In case of corticosterone measurements for AIS and AIS+10d, Kruskal–Wallis test followed by Mann–Whitney *post-hoc* test with Bonferroni's adjustment was used because the data distributions called for a non-parametric analysis. For the analysis of the BDNF ELISA data, ANOVA followed by *post-hoc* Fisher's least significant difference (LSD) test was used for pairwise comparison in the case of AIS and AIS+10d. P-values less than 0.05 were considered significant.

## Results

### Chronic stress elicits opposite effects on BDNF levels in the BLA and area CA3

As described above, chronic immobilization stress (CIS; 2 h/day for 10 days) leads to contrasting patterns of dendritic remodeling in the BLA versus hippocampal area CA3. Specifically, 1 day after the termination of stress, the same CIS that caused dendritic atrophy in the CA3 pyramidal neurons, elicited dendritic growth and spinogenesis in the BLA principal neurons. As BDNF promotes dendritic and spine growth [18], we reasoned that the levels of BDNF expression may reflect the divergent effects of chronic stress on neuronal morphology in the BLA versus area CA3. To test this prediction, we conducted ELISA analysis on tissue micro-dissected from these two brain regions in control and CIS treated rats (Figure 1A). There was significantly lower levels of BDNF protein in the area CA3 1 day after chronic stress (Control, 1.00±0.11; n = 5; CIS+1d, 0.43±0.07; data normalized to control animals; n = 5, P<0.05; Figure 1B). In striking contrast, the same chronic stress led to significantly higher levels of BDNF protein in the BLA 1 day later (Control, 1.00±0.01; n = 6; CIS+1d, 1.44±0.15; data normalized to control animals; n = 8; P*<*0.05; Figure 1C). Thus, these data indicate that similar to its differential effects on dendritic arborization, CIS also modulates BDNF levels in the area CA3 and BLA in contrasting fashion.

We also confirmed the efficacy of our CIS paradigm by assessing two widely used indicators of stress. First, we measured, 1 day after the end of the 10 day CIS protocol, the blood serum levels of corticosterone, a common indicator of HPA axis activation. There was a significant increase in the levels of corticosterone in CIS animals relative to control animals (Control, 83.8±42.2 ng/ml of serum; n = 5; CIS+1d, 338.2±94.8 ng/ml of serum; n = 6; P<0.05; Figure 1D). Second, we compared changes in body weight (Table 1) and found a significant reduction in the percentage weight gained 1 day after CIS (Control, 30.26±1.37%; n = 12; CIS+1d, 14.4±1.94%; n = 12; P<0.05).

**Table 1:** Summary of body weight measures.

*Experimental group*	*Control*		*Stress*		*p value*
	*Mean ± SEM*	*n*	*Mean ± SEM*	*n*	
CIS	30.3±1.4%	12	14.4±1.9%	12	0.00*
CIS+21d	73.4±4.1%;	4	70.0±3.5%	6	0.54
AIS	1.3±0.3%	6	0.9±0.4%	5	0.38
AIS+10d	30.3±1.4%	12	17.4±5.4%;	6	0.07

Data are presented as mean ± SEM. Significant differences.

*, p<0.05; Unpaired, two-tailed, Student's t-test was used to compare stress and control groups.

### Recovery after chronic stress fails to reverse elevated levels of BDNF in the BLA, but not in area CA3

Earlier studies have reported that the impact of chronic stress also differs between the hippocampus and amygdala in terms of its temporal persistence. For instance, dendritic hypertrophy in the BLA persists even after 21 days of stress-free recovery following 10 days of CIS. On the other hand, CIS-induced hippocampal CA3 atrophy is reversible within the same 21-day period of recovery. Since the data presented so far indicates that the CIS-induced increase and decrease in BDNF levels parallels BLA hypertrophy and CA3 atrophy respectively, we tested if the temporal profile of BDNF levels during post-stress recovery also differ in the two regions. Twenty one days after the end of CIS, we found no difference in BDNF levels in area CA3 between control and CIS animals (Control, 1.00±0.15; n = 6; CIS+21d, 1.00±0.2; data normalized to control animals; n = 6; P>0.05; Figure 2B). In the BLA, by contrast, BDNF levels remained significantly elevated even after the same post-CIS recovery period (Control, 1.00±0.02; n = 4; CIS+21d, 2.3±0.49; data normalized to control animals; n = 6; P<0.05; Figure 2C). Thus, unlike CA3 area, CIS-induced up-regulation of BDNF protein levels in the BLA persists up to 21 days after the end of stress.

We also monitored the impact of CIS on blood serum levels of corticosterone and relative gain in body weight after the same recovery period. Corticosterone levels remained elevated even 21 days after CIS (Control, 43.0±11.7 ng/ml of serum; n = 7; CIS+21d, 146.3±34.9 ng/ml of serum; n = 6; P<0.05; Figure 2D). In contrast, there was no difference in the percentage gain in body weight between stressed and unstressed animals after the recovery period (Control, 73.41±4.1%; n = 4; CIS+21d, 70.0±3.5% n = 6 P>0.05) (Table 1).

### Acute stress also differs in its impact on BDNF expression over time in the BLA and area CA3

The results described so far show that BDNF levels in area CA3 and BLA, following exposure to CIS, are strikingly different both in terms of the direction and time course of change. In contrast to the robust effects seen when the 2 h immobilization stress is repeated for 10 consecutive days, only a single episode of the same stress (acute immobilization stress or AIS) is known to trigger a different temporal pattern of changes in the BLA. Unlike CIS, AIS fails to affect spine density or dendritic arborization, when measured 1 day later. However, the same AIS leads to a gradual increase in spine density 10 days later in the BLA. Therefore, next we examined if AIS modulates BDNF levels in the hippocampus and amygdala over time. We found only a modest reduction in BDNF in area CA3 that was not statistically significant either 1 or 10 days after AIS (Control, 1.00±0.11; n = 9; AIS+1d, 0.73±0.05; data normalized to control animals; n = 9; AIS+10d, 1.02±0.19; data normalized to control animals; n = 6; P>0.05; Figure 3B). However, the same AIS caused a significant increase in BDNF levels in the BLA 1 day later (Control, 1.00±0.06; n = 6; AIS+1d, 2.31±0.24; data normalized to control animals; n = 5; P<0.05; Figure 3C). Moreover, this AIS-induced elevation in BDNF levels persisted in the BLA even after 10 days (CTR, 1.00±0.06; n = 6; AIS+10d, 1.98±0.46; data normalized to control animals; n = 6; P<0.05; Figure 3C). These findings demonstrate that even a shorter duration of stress triggers changes in BDNF in the BLA and CA3 area that differ considerably in their direction and temporal features.

Since the AIS paradigm is expected to be less severe compared to CIS, we also monitored its impact on serum levels of corticosterone and body weight gain at the same time points after AIS when BDNF protein levels were measured. Relative to controls, AIS caused a significant increase in corticosterone levels 1 day later (Control, 83.8±26.0 ng/ml of serum; n = 11, AIS+1d, 594.7±221.2 ng/ml of serum n = 5 P<0.05; Figure 3D). 10 days after AIS, corticosterone levels continued to remain high, although the difference was no longer statistically significant (Control, 83.8±26.0 ng/ml of serum; n = 11; AIS+10d, 232.9±78.9 ng/ml of serum; n = 6; P>0.05). Further, unlike CIS, AIS did not cause any significant change in the percentage of body weight gain 1 day later (Control, 1.31±0.31%; n = 6; AIS+1d, 0.93±0.41%; n = 5; P>0.05) (Table 1). This absence of any effect on body weight was also seen after 10 days (Control, 30.26±1.37%; n = 12; AIS + 10d, 17.37±5.39%; n = 6; P>0.05) (Table 1). Taken together, it is only 1 day after CIS that we observed a significant effect of stress on body weight gain.

## Discussion

This study explored two key facets of stress-induced modulation of BDNF expression in the hippocampus and amygdala – one in terms of region-specific differences, and the other in the temporal domain. Because BDNF regulates dendritic architecture and spines, both major targets of stress-induced structural plasticity, we hypothesized that the levels of BDNF expression would reflect the divergent effects of stress on the hippocampus and amygdala. To test this hypothesis, we used two very different paradigms of immobilization stress – an acute paradigm involving a single 2 h session (AIS) and a chronic version wherein the same 2-hour stress was repeated for 10 consecutive days (CIS). First, we tested whether chronic stress elicits changes in BDNF protein levels that parallel the contrasting patterns of dendritic remodeling observed previously in the amygdala and hippocampus. We report that the same CIS has strikingly opposite effects on BDNF expression one day after the end of CIS – it reduces BDNF in area CA3, while it increases BDNF in the BLA. This contrasting modulation was accompanied by a significant up-regulation in circulating corticosterone levels. Second, in light of earlier reports on the unique temporal features of structural plasticity elicited in the amygdala by both chronic and acute stress, we tested whether changes in BDNF levels also exhibit distinct patterns across time in the two areas. We find that not only does CIS elevate BDNF levels in the BLA, but this increase lasts for at least 21 days after the end of CIS, which is consistent with earlier findings on CIS-induced dendritic hypertrophy in the BLA persisting for the same duration after stress. In area CA3, however, CIS-induced decrease in BDNF levels reverses to normal levels within the same post-stress period of 21 days. This in turn is consistent with the previously reported reversal of CA3 dendritic atrophy over the same time frame. However, levels of corticosterone remain elevated even after 21 days of recovery from stress. Finally, even acute immobilization stress (AIS) modulates BDNF expression differentially in the two brain areas. Exposure to AIS caused a trend in lower BDNF levels in the CA3 area one day later, but neither was this decrease statistically significant nor did it last for 10 days post-stress. In contrast, the same AIS caused a more robust increase in BDNF levels in the BLA that remained significantly above control levels even 10 days after AIS. Interestingly, according to an earlier study, AIS led to a delayed increase in BLA spine-density that was manifested 10 days, but not 1 day after AIS. However, we find the highest levels of BDNF in the BLA 1 day after AIS. Ten days after AIS, the BLA continues to express significantly higher levels of BDNF, albeit at levels that are lower than the 1-day time point. Thus, AIS appears to trigger a rise in BDNF relatively soon after stress that precedes the gradual build-up in spine-density in the BLA. Future studies will be necessary to examine if this initial peak in BDNF levels serves as an early signal for plasticity mechanisms that eventually culminates in delayed BLA spinogenesis 10 days later.

The contrasting effects of stress on BDNF shed new light on earlier findings on the differential patterns of cellular changes elicited by chronic and acute stress in the amygdala versus hippocampus. Both in terms of the direction and temporal profile of these changes, the enhanced levels of BDNF elicited by chronic stress parallels the profile of dendritic growth and spinogenesis in the BLA. These findings are also significant in view of an earlier study demonstrating that transgenic overexpression of BDNF enhances spine-density in the BLA of mice [16]. BLA spinogenesis is also elicited by chronic stress. Importantly, transgenic overexpression of BDNF occludes chronic stress induced spinogenesis in the BLA. Together these findings suggest a role for BDNF in stress-induced structural plasticity in the amygdala. The results reported here also add to the earlier studies on region-specific differences showing stress-induced increase in BDNF expression in the hypothalamus [19] and the nucleus acumbens (NAc) [20] compared to decreased levels in the hippocampus. Further, the up-regulation of BDNF seen in the NAc after social-defeat stress persists for as long as 4 weeks, similar to the prolonged increase we see in the BLA [20]. The upregulation of BDNF reported earlier in the hypothalamic paraventricular nucleus occurs in response to both single 2 h as well as chronic 7 days of immobilization stress [19]. On the other hand, chronic stress-induced reduction of BDNF levels, and its reversal during stress-free recovery, mirrors earlier findings on reversible dendritic atrophy elicited by chronic stress in hippocampal area CA3. However, short episodes of acute stress lasting 15 min or 60 min induces a transient increase in BDNF mRNA levels in the hippocampus which is in sharp contrast to the decrease in BDNF mRNA expression seen in response to longer stress [21]. Together, these findings also highlight the importance of investigating the potential mechanisms that cause the same stress to have opposite effects on BDNF expression as well as the differential impact of variable durations of stress on BDNF levels.

Exposure to stressful events leads to glucocorticoid release by the activation of the hypothalamic–pituitary–adrenal (HPA) axis. The temporal profile of changes in corticosterone profile shows an initial rise 1 day after AIS which decreases 10 days later and returns to levels that are not significantly different from control levels. Thus, the variations in corticosterone levels reported here appear to depend on both the severity of the stress paradigm and time points of measurement after the termination of stress. Our observations are broadly consistent with earlier reports showing that the output of the HPA axis is influenced by the periodicity, intensity and degree of habituation of the stressor [22]–[26]. The reduction in hippocampal BDNF due to acute and chronic stress, in turn, is known to be mediated at least in part by stress-induced increase in glucocorticoids [10], [27]. Yet the same increase in corticosterone appears to be associated with enhanced BDNF levels in the BLA. Stress also leads to significant increase in extracellular levels of glutamate in the hippocampus and amygdala [28]–[30]. This suggests that although some of the immediate consequences of stress – elevated glucocorticoids and glutamate – are similar in both brain areas, they subsequently lead to contrasting patterns of BDNF expression and structural plasticity. This implies that signaling mechanisms more downstream of the initial changes in glucocorticoids and glutamate, but upstream of BDNF, may hold the key to the differential impact of stress in these brain areas. Importantly BDNF infusion into the hippocampus of stressed rodents helped to protect against the deleterious effects of stress despite high levels of circulating corticosterone [31]. This suggests that BDNF could be a final point of convergence for the stress induced effects in the hippocampus. BDNF-mediated signaling is involved in stress response but the direction and nature of signaling is region-specific, stress specific and is influenced by epigenetic modifications along with post translational modifications [32]. Thus, brain region specific variations in BDNF expression is a key question that requires further investigation. Moreover, a complex balance is maintained in BDNF-driven neuronal plasticity, based on the competition between BDNF-TrkB system and pro-BDNF-p75 system [33]. Interestingly, activation of TrkB and p75NTR promote and suppress dendritic spine growth respectively [33]. Since the findings presented here do not distinguish between TrkB and p75NTR, which are known to have opposing biological functions, future studies will be necessary to elucidate the impact of stress on these different forms in different brain regions.

What may be the functional consequences of the divergent effects of stress on BDNF in the hippocampus and amygdala? Growing evidence has linked growth of dendrites and spines in the BLA – caused either by stress or BDNF overexpression – to enhanced anxiety-like behavior [8], [15]. This is relevant in light of our results showing that the same time points when stress triggers higher anxiety is also when BDNF is elevated in the BLA. For instance, both anxiety and BDNF are increased 1 and 21 days after CIS. Interestingly, BDNF mRNA has been shown to be elevated transiently in the BLA 2 hours after cued fear conditioning and such a temporally restricted elevation of BDNF signaling is believed to be a key step in the formation of a cue-specific fear [34], [35]. In contrast, a more sustained up-regulation of BDNF in the BLA may underlie the pathological increase in fear and anxiety observed following stress [16]. Hippocampal memory and synaptic plasticity also depend on BDNF, and reduced levels of BDNF following chronic stress is consistent with a large body of evidence showing impairment of hippocampal function and loss of hippocampal volume caused by stress [36]–[39]. Further as hippocampal BDNF is critically involved in resilience to stress, any reduction can enhance vulnerability to stress [40]. Therefore, the opposite effects on BDNF in the amygdala and hippocampus may provide a molecular basis for the contrasting behavioral effects of stress on memories encoded by these two brain areas. Moreover, these two brain areas differ not only in their response to stress, but also in how they regulate the stress response. While the hippocampus exerts a negative feedback regulation of the stress response via the HPA axis, the amygdala has the opposite effect [41]. Therefore, the differential effects on BDNF in these two brain regions following stress could lead to an imbalance in HPA axis function through a gradual loss of hippocampal inhibitory control as well as a gain in excitatory control exerted by the amygdala. The regional differences in the pattern of BDNF expression triggered by stress also pose a significant challenge for pharmacological interventions aimed at countering the effects of stress on the amygdala and hippocampus. Therefore, elucidation of the mechanisms behind the differential effects of stress on BDNF in the hippocampus and amygdala is likely to provide useful insights into novel therapeutic interventions against stress-related psychiatric disorders that are characterized by impaired cognitive function and abnormally high fear and anxiety.
